# Supplementary material for: Physical activity for people living with cancer: Knowledge, attitudes, and practices of general practitioners in Australia
Source: PLoS One. 2020 Nov 9;15(11):e0241668. doi: 10.1371/journal.pone.0241668 (PMC7652282; doi:10.1371/journal.pone.0241668)
Supplement: S1 Appendix — (DOCX) [file pone.0241668.s002.docx]

**S1 Appendix - Survey**

Physical activity and cancer patients- General practitioners views

**Participant Information Form**

**Project Title** Knowledge, Attitudes and Practice of General Practitioners in the Promotion of Physical Activity for Cancer Patients: A National Survey

**Primary Researcher**

Name: Georgina Alderman
Faculty: Health
Phone: 0424795171
Email: u3144356@uni.canberra.edu.au

**Supervisors:**

Name: Kellie Toohey
Phone: +61 (0)2 6206 8873
Email: Kellie.Toohey@canberra.edu.au

Name: Stuart Semple
Phone: +61 2 62012855
Email: stuart.semple@canberra.edu.au

**Primary aim:** - To investigate General Practitioners knowledge, attitudes and practice in the promotion of physical activity for cancer patients

**Secondary aim:** - To determine if general practitioners individual physical activity levels impact rate of referral - To understand if general practitioners are likely to refer cancer patients during or post cancer (chemotherapy, radiation and immunotherapy) treatment to participate in physical activity

**Participant Involvement**

Participants who agree to participate in the research will be asked to:

1. Provide informed consent

2. Conduct a 5-7 minute survey

*Note: Once clicking SUBMIT on the online version you will be unable to withdraw from the survey.*

**Confidentiality** Only the researcher/s will have access to the individual information provided by participants. Privacy and confidentiality will be assured at all times. The research outcomes may be presented at conferences and written up for publication. However, in all these publications, the privacy and confidentiality of individuals will be protected.

**Data Storage** The information collected will be stored securely on a password protected computer throughout the project and then stored at the University of Canberra for the required five year period after which it will be destroyed according to university protocols.

**Ethics Committee Clearance** The project has been approved by the Human Research Ethics Committee of the University of Canberra (HREC – 20191802).

**Queries and Concerns**   Queries or concerns regarding the research can be directed to the researcher and/or supervisor. Their contact details are at the top of this form. You can also contact the University of Canberra’s Research Ethics & Integrity Unit. You can either contact Mr Hendryk Flaegel via phone 02 6201 5220, Ms Maryanne Simpson via phone 02 6206 3916 or email humanethicscommittee@canberra.edu.au.

*Note:* Survey has been adapted from the following studies: - Daley, A., Bowden, S., Rea, D., Billingham, L., & Carmicheal, A. (2008). What advice are oncologists and surgeons in the United Kingdom giving to breast cancer patients about physical activity? *International Journal of Behavioral Nutrition and Physical Activity, 5*(46). doi:10.1186/1479-5868-5-46 - Fisher, A., Williams, K., Beeken, R., & Wardle, J. (2015). Recall of physical activity advice was associated with higher levels of physical activity in colorectal cancer patients. *BMJ Open, 5*(e006853). doi:10.1136/bmjopen-2014- 006853 - Hardcastle, S., Kane, R., Chivers, P., Hince, D., Dean, A., Higgs, D., & Cohen, P. (2018). Knowledge, attitudes, and practice of oncologists and oncology health care providers in promoting physical activity to cancer survivors: an international survey. *Supportive Care in Cancer, 26*, 3711-3719. doi:https://doi.org/10.1007/s00520-018-4230-1 - Haussmann, A., Ungar, N., Gabrian, M., Tsiouris, A., Sieverding, M., Wiskemann, J., & Steindorf, K. (2018). Are healthcare professionals being left in the lurch? The role of structural barriers and information resources to promote physical activity to cancer patients. *Supportive Care in Cancer, 26*(1), 4087–4096. doi:https://doi.org/10.1007/s00520-018-4279-x - Jones, L., Courneya, K., Peddle, C., & Mackey, J. (2005). Oncologists’ opinions towards recommending exercise to patients with cancer: a Canadian national survey. *Support Care Cancer, 13*, 929-937. doi:10.1007/s00520-005-0805-8 - Nadler, M., Bainbridge, D., Tomasone, J., Cheifetz, O., Juergens, R., & Sussman, J. (2017). Oncology care provider perspectives on exercise promotion in people with cancer: an examination of knowledge, practices, barriers, and facilitators. *Support Care Cancer, 25*, 2297-2304. doi:10.1007/s00520-017-3640-9 - Park, J., Oh, M., Yoon, Y., Lee, C., Jones, L., Kim, S., . . . Jeon, J. (2015). Characteristics of attitude and recommendation of oncologists toward exercise in South Korea: a cross sectional survey study. *BMC Cancer, 15*(249). doi:10.1186/s12885-015-1250-9

**Informed Consent**

Welcome to the research study! We are interested in understanding the 'Knowledge, Attitudes and Practice of General Practitioners in the Promotion of Physical Activity for Cancer Patients'. You will be presented with information relevant to Physical Activity and Cancer patient and/or survivor involvement and asked to answer some questions about it. Please be assured that your responses will be kept completely confidential.  

 By clicking the button below, you acknowledge that your participation in the study is voluntary, you are over 18 years of age, and that you are aware that you may choose to terminate your participation in the study at any time and for any reason.

 Please note that this survey will be best displayed on a laptop or desktop computer.  Some features may be less compatible for use on a mobile device.

- I consent, begin the study (1)
- I do not consent, I do not wish to participate (2)

End of Block: Informed Consent

Start of Block: Define Physical Activity

0 This survey will question you on physical activity. 
 
**Definition of Physical Activity**
Physical activity is an umbrella term defined as any bodily movement produced by skeletal muscles that requires energy expenditure. Structured exercise includes any planned, structured, repetitive and intentional movement intended to improve or maintain physical fitness. Exercise is a subcategory of physical activity.  

End of Block: Define Physical Activity

Start of Block: Answer the following questions in relation to ‘DURING’ cancer treatment:

Answer the following questions in relation to '**DURING'** cancer treatment


**For the purposes of this study "during treatment" refers to during chemotherapy, radiation or immunotherapy treatment*

Q1 For the following statements, please select your response along the scale of 1 (strongly disagree) to 7 (strongly agree)

|  | Strongly Disagree | Disagree | Slightly Disagree | Slightly Agree | Agree | Strongly Agree |
| --- | --- | --- | --- | --- | --- | --- |

|  | 1 | 2 | 3 | 4 | 5 | 6 | 7 |
| --- | --- | --- | --- | --- | --- | --- | --- |

| In my opinion, physical activity can be **beneficial** during a cancer treatment () | 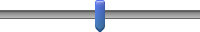 |
| --- | --- |
| In my opinion, physical activity can be **important** during a cancer treatment () | 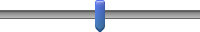 |
| In my opinion, physical activity can be **safe** during cancer treatment () | 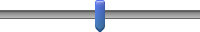 |
| Most patients believe they **should** be physically activity during cancer treatment () | 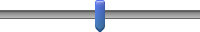 |
| Most fellow General Practitioners think patients **should** participate in physical activity during cancer treatment () | 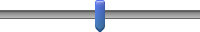 |
| Most of my patients are **capable** of participating in physical activity during cancer treatment () | 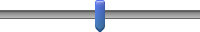 |
| I believe **high intensity** physical activity is a contraindication for patients **during** treatment () | 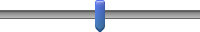 |

End of Block: Answer the following questions in relation to ‘DURING’ cancer treatment:

Start of Block: Please answer the following questions in relation to Post Treatment patients

Please answer the following questions in relation to **'POST'** cancer treatment **For the purposes of this survey "post treatment" means post chemotherapy, radiation or immunotherapy treatment.*

Q2 For the following statements, please select your response along the scale of 1 (strongly disagree) to 7 (strongly agree)

|  | Strongly Disagree | Disagree | Slightly Disagree | Slightly Agree | Agree | Strongly Agree |
| --- | --- | --- | --- | --- | --- | --- |

|  | 1 | 2 | 3 | 4 | 5 | 6 | 7 |
| --- | --- | --- | --- | --- | --- | --- | --- |

| I feel **confident** in giving general advice to cancer patients about physical activity () | 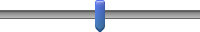 |
| --- | --- |
| Discussing physical activity with cancer patients is part of my **role** as a general practitioner () | 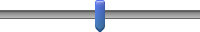 |
| Other general practitioners believe it is part of their **role** to discuss physical activity with their patients () | 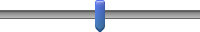 |
| I believe **high intensity** physical activity is a contraindication for patients **post** treatment () | 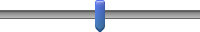 |

End of Block: Please answer the following questions in relation to Post Treatment patients

Start of Block: Knowledge of Physical Activity

**Knowledge of Physical Activity**

Q3 Which of the following recommendations do you believe to be the **target** for generalised physical fitness?

- 50 minutes moderate intensity exercise or 25 minutes high intensity exercise per week (1)
- 100 minutes moderate intensity exercise or 50 minutes high intensity exercise per week (2)
- 150 minutes moderate intensity exercise or 75 minutes high intensity exercise per week (3)
- 300 minutes moderate intensity exercise or 150 minutes high intensity exercise per week (4)

Q4 For the following questions please select either YES or NO:

|  | Yes (1) | No (2) |
| --- | --- | --- |
| During your **studies** did you ever receive training on the role of physical activity for cancer patients? (1) |  |  |
| Have you completed any **additional training** or courses on physical activity or exercise advice? (2) |  |  |
| Are you aware of the **Clinical Oncology Society of Australia's** (COSA's) position statement in regards to physical activity for cancer patients? (4) |  |  |
| Do Physical activity guidelines for general population **differ** to those for the cancer population? (5) |  |  |

Q5 For the following statements, please select your response along the scale of 1 (strongly disagree) to 7 (strongly agree)

|  | Strongly Disagree | Disagree | Slightly Disagree | Slightly Agree | Agree | Strongly Agree |
| --- | --- | --- | --- | --- | --- | --- |

|  | 1 | 2 | 3 | 4 | 5 | 6 | 7 |
| --- | --- | --- | --- | --- | --- | --- | --- |

| Current evidence suggests that **regular** physical activity can improve the **quality of life** of cancer patients () | 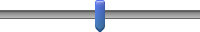 |
| --- | --- |
| Current evidence suggests **regular** physical activity is associated with reduced **negative side effects** of cancer treatment () | 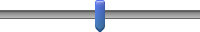 |

End of Block: Knowledge of Physical Activity

Start of Block: Promotion of Physical Activity

**Promotion of Physical Activity**

Q6 What percentage of your cancer patients have you recommended physical activity to?

- 0% (1)
- 1-20% (2)
- 21-40% (3)
- 41-60% (4)
- 61-80% (5)
- 81-100% (6)

Q7 Of the following physical activity modalities, rank in order of preference (click and drag into order of preference):

______ Cardiovascular (i.e. swimming, jogging, tennis, gardening etc.) (1)

______ Weight/ resistance training (i.e. individualised gym based program, resistance band home exercise program etc.) (2)

______ Stretching (3)

______ Walking (4)

______ Pelvic floor exercises (5)

______ Pilates (6)

______ Yoga (7)

Q8 For the following statements, please select your response along the scale of 1 (strongly disagree) to 7 (strongly agree)

|  | Strongly Disagree | Disagree | Slightly Disagree | Slightly Agree | Agree | Strongly Agree |
| --- | --- | --- | --- | --- | --- | --- |

|  | 1 | 2 | 3 | 4 | 5 | 6 | 7 |
| --- | --- | --- | --- | --- | --- | --- | --- |

| My patients are amenable to receiving **advice** on the importance of increasing their physical activity levels () | 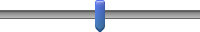 |
| --- | --- |
| I believe that patients would **follow** my advice, if I provided physical activity recommendations () | 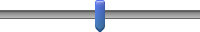 |
| My patients **ask** me about physical activity recommendations () | 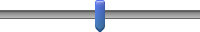 |
| For me, providing a recommendation is **easy** () | 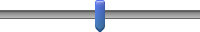 |

End of Block: Promotion of Physical Activity

Start of Block: Referring patients:

**Referring Patients**

Q9 What percentage of your cancer patients do you refer to participate in an exercise program or to receive physical activity advice?

- 0% (1)
- 1-20% (2)
- 21-40% (3)
- 41-60% (4)
- 61-80% (5)
- 81-100% (6)

Q10 Are you more likely to refer cancer patients currently going through treatment or cancer patients post treatment to participate in physical activity? (Treatment referring to chemotherapy, radiation or immunotherapy)

- Patients currently going through treatment (1)
- Patients post treatment (2)
- Both equally (3)
- Neither (4)

Q11 Do you have access to resources to give to your patients in regards to physical activity recommendations and potential services?

- Yes (1)
- No (2)

Q12 Rank in order of preference the allied health care practitioner you feel would be most suited to provide physical activity guidance (click and drag in order of preference)

______ Physiotherapist (1)

______ Exercise physiologist (2)

______ Personal trainer (3)

______ Occupational therapist (5)

______ Sports medicine doctor (6)

______ Sport scientist (10)

End of Block: Referring patients:

Start of Block: Personal Physical Activity Levels

**Personal Physical Activity Levels**

Q13 Do you participate in any structured exercise on a regular bases (>2 times per week)? (i.e. gym based programs, jogging, cycling etc.)

- Yes (4)
- No (5)

Q14 What best describes your activity level?

- Vigorously active for at least 30 min, 3 times per week (1)
- Moderately active at least 3 times per week (2)
- Seldomly active, preferring sedentary activities (3)

End of Block: Personal Physical Activity Levels

Start of Block: Demographic Information

Q15 What is your age? (In years)

________________________________________________________________

Q16 What gender do you identify as?

- Male (1)
- Female (2)
- Other (please specify) (3) ________________________________________________
- Prefer not to say (4)

Q17 How many **years** have you been practicing as a General Practitioner?

________________________________________________________________

Q18 The location of the practice that I currently work at is classified as:

- Urban (1)
- Sub-Urban (2)
- Rural (3)

Q19 Within the last 12 months have you consulted a cancer patient in your capacity as a general practitioner?

- Yes (1)
- No (2)

End of Block: Demographic Information
