# Supplementary material for: Physical activity for people living with cancer: Knowledge, attitudes, and practices of general practitioners in Australia
Source: PLoS One. 2020 Nov 9;15(11):e0241668. doi: 10.1371/journal.pone.0241668 (PMC7652282; doi:10.1371/journal.pone.0241668)
Supplement: S2 Appendix — (DOCX) [file pone.0241668.s003.docx]

**S2 Appendix-** Test-retest analysis procedure

Likehert-

|  | Pearson Correlation | Sig. (2-tailed) (p-value) | Coefficient of determination |
| --- | --- | --- | --- |
| During treatment | .997 | .000 | 99.40% |
| Post treatment | .982 | .000 | 96.43% |
| Evidence based practice | .984 | .000 | 96.83% |
| Promotion of PA | .983 | .000 | 96.63% |

A Pearson’s product-moment correlation was run to assess the relationship between likehert construct responses in initial response and secondary retest responses. During treatment, post treatment, evidence based practice and promotion of physical activity constructs all presented a strong correlation from general practitioners initial response and retest response 2 weeks later. Coefficients of determination were presented as 99.40, 96.43, 96.83 and 96.63% respectively. Preliminary analyses presents a linear relationship between both variables in all constructs (p>0.05), there were no outliers.

Yes/no questions- Sign test

|  | Null Hypothesis | Test | Sig. | Decision |
| --- | --- | --- | --- | --- |
| 4A | The median of differences between T1 and T2 = 0 | Related- samples Sign Test | 1.000 | Retain the null hypothesis |
| 4B | The median of differences between T1 and T2 = 0 | Related- samples Sign Test | 1.000 | Retain the null hypothesis |
| 4C | The median of differences between T1 and T2 = 0 | Related- samples Sign Test | 1.000 | Retain the null hypothesis |
| 4D | The median of differences between T1 and T2 = 0 | Related- samples Sign Test | 1.000 | Retain the null hypothesis |
| Resource access | The median of differences between T1 and T2 = 0 | Related- samples Sign Test | 1.000 | Retain the null hypothesis |
| Personal structured PA | The median of differences between T1 and T2 = 0 | Related- samples Sign Test | 1.000 | Retain the null hypothesis |
| Cancer patient frequency | The median of differences between T1 and T2 = 0 | Related- samples Sign Test | 1.000 | Retain the null hypothesis |

An exact sign test was used to compare the differences in responses in Yes/ No questions within this survey. Initial response was compared to secondary retest response where no statistical difference was noticed between responses. When manually tallying the varied inter-person response between all 10 respondents, 0 changes in response were evident. This presents strong validity for the yes/no questions.

Ordinal-

|  | Spearman’s rho | Sig. (2-tailed) (p-value) |
| --- | --- | --- |
| PA guidelines | 1.000 | .000 |
| % Recommended PA | .978 | .000 |
| % Referral | 1.000 | .000 |
| Who to refer to | .933 | .000 |
| Personal PA levels | 1.000 | .000 |
| Age | 1.000 | .000 |
| Gender | 1.000 | .000 |
| Years practicing | 1.000 | .000 |
| Location | 1.000 | .000 |

A Pearson’s product-moment correlation was run to assess the relationship between categorical questions responses in initial response and secondary retest responses. No variations in responses were evident other than two questions. “What percentage of your cancer patients have you recommended physical activity to?” and “Are you more likely to refer cancer patients currently going through treatment or post treatment to participate in physical activity?” (Including options of both equally or neither in addition). Preliminary analysis showed the relationship to be monotonic, as assessed by visual review of a scatterplot. Results were statistically significant for both questions, presenting a strong positive correlation between responses, *r_s_*(98) = .948-1.000, p<0.0005.

Scalar-

| **Question 7** | Spearman’s rho | Sig (2-tailed) |
| --- | --- | --- |
| Cardiovascular | 1.000 | .000 |
| Weight/ resistance training | 1.000 | .000 |
| Stretching | 1.000 | .000 |
| Walking | 1.000 | .000 |
| PF exercises | 1.000 | .000 |
| Pilates | 1.000 | .000 |
| Yoga | 1.000 | .000 |

| **Question 12** | Spearman' rho | Sig (2-tailed) |
| --- | --- | --- |
| Physiotherapist | 1.000 | .000 |
| Exercise physiologist | 1.000 | .000 |
| Personal trainer | .952 | .000 |
| Occupational therapist | .967 | .000 |
| Sports medicine doctor | .948 | .000 |
| Sport scientist | 1.000 | .000 |

A Spearman’s rank-order correlation was run to assess the relationship between initial response and secondary retest response for the scalar questions of the survey (questions 7 and 12). Preliminary analysis showed the relationship to be monotonic, as assessed by visual review of a scatterplot. Results were statistically significant for both questions, presenting a strong positive correlation between both responses, *r_s_*(98) = .948-1.000, p<0.0005.
