# Supplementary figures and images for: Physical activity for people living with cancer: Knowledge, attitudes, and practices of general practitioners in Australia
Source: PLoS One. 2020 Nov 9;15(11):e0241668. doi: 10.1371/journal.pone.0241668 (PMC7652282; doi:10.1371/journal.pone.0241668)

**S3 Appendix - PCA total variance SPSS output**


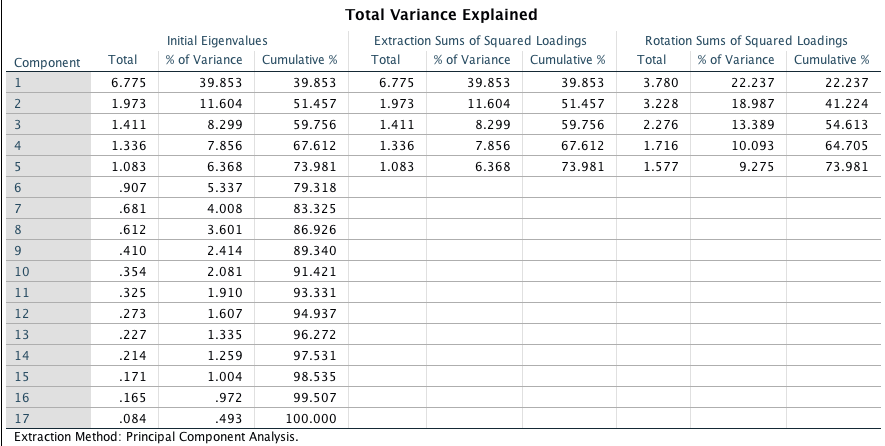

Supplement: S3 Appendix — (DOCX) [file pone.0241668.s004.docx]
